# Influenza Vaccination Guidelines and Vaccine Sales in Southeast Asia: 2008–2011

**DOI:** 10.1371/journal.pone.0052842

**Published:** 2012-12-21

**Authors:** Vinay Gupta, Fatimah S. Dawood, Charung Muangchana, Phan Trong Lan, Anonh Xeuatvongsa, Ly Sovann, Remigio Olveda, Jeffery Cutter, Khin Yi Oo, Theresia Sandra Diah Ratih, Chong Chee Kheong, Bryan K. Kapella, Paul Kitsutani, Andrew Corwin, Sonja J. Olsen

**Affiliations:** 1 Columbia University, College of Physicians and Surgeons, New York, New York, United States of America; 2 Thailand Ministry of Public Health – U.S. CDC Collaboration, Nonthaburi, Thailand; 3 Influenza Division, National Center for Immunization and Respiratory Diseases, Centers for Disease Control and Prevention, Atlanta, Georgia, United States of America; 4 National Vaccine Committee Office, Department of Disease Control, Ministry of Public Health, Nonthaburi, Thailand; 5 General Department of Preventive Medicine, Ministry of Health, Hanoi, Socialist Republic of Vietnam; 6 Expanded Program on Immunizations, Ministry of Public Health, Vientiane, Lao People’s Democratic Republic; 7 CDC Department, Ministry of Public Health, Phnom Penh, Cambodia; 8 Research Institute for Tropical Medicine, Department of Health, Muntinlupa City, Philippines; 9 Communicable Diseases Division, Ministry of Health, Singapore; 10 National Health Laboratory, Ministry of Health, Nay Pyi Taw, The Republic of the Union of Myanmar; 11 Expanded Program on Immunizations, Ministry of Public Health, Jakarta, Republic of Indonesia; 12 Disease Control Division, Ministry of Health, Kuala Lumpur, Malaysia; University of Oxford, Viet Nam

## Abstract

**Background:**

Southeast Asia is a region with great potential for the emergence of a pandemic influenza virus. Global efforts to improve influenza surveillance in this region have documented the burden and seasonality of influenza viruses and have informed influenza prevention strategies, but little information exists about influenza vaccination guidelines and vaccine sales.

**Methods:**

To ascertain the existence of influenza vaccine guidelines and define the scope of vaccine sales, we sent a standard three-page questionnaire to the ten member nations of the Association of Southeast Asian Nations. We also surveyed three multinational manufacturers who supply influenza vaccines in the region.

**Results:**

Vaccine sales in the private sector were <1000 per 100,000 population in the 10 countries. Five countries reported purchasing vaccine for use in the public sector. In 2011, Thailand had the highest combined reported rate of vaccine sales (10,333 per 100,000). In the 10 countries combined, the rate of private sector sales during 2010–2011 (after the A(H1N1)2009pdm pandemic) exceeded 2008 pre-pandemic levels. Five countries (Indonesia, Malaysia, Singapore, Thailand and Vietnam) had guidelines for influenza vaccination but only two were consistent with global guidelines. Four recommended vaccination for health care workers, four for elderly persons, three for young children, three for persons with underlying disease, and two for pregnant women.

**Conclusions:**

The rate of vaccine sales in Southeast Asia remains low, but there was a positive impact in sales after the A(H1N1)2009pdm pandemic. Low adherence to global vaccine guidelines suggests that more work is needed in the policy arena.

## Introduction

Influenza is one of the world’s most prevalent vaccine preventable diseases, responsible for three to five million cases of severe illness and 250 to 500 thousand deaths annually [1]. Over the past 10 years, there has been an increased focus on establishing more robust surveillance for influenza viruses globally with a heavy emphasis on tropical and subtropical Southeast Asia. Resources, including more than $1 billion from the United States government since 2005 [2], have been preferentially applied to Southeast Asia because of the emergence and persistence of avian influenza A (H5N1) and concern that the region is a “hotspot” for the evolution of other influenza viruses [3]. As demonstrated by H5N1 and the 2009 pandemic caused by the A(H1N1)2009pdm virus, investing in prevention and preparedness strategies is essential for all countries in the region. Moreover, given the increasing trend toward urbanization in Asia [4], the resulting evolution of mega cities, and the ease of modern air travel, the spread of a lethal pandemic influenza virus is a very real possibility and cause for continued concern.

Until recently, use of seasonal influenza vaccine was extremely uncommon in Southeast Asia. For example, doses were primarily limited to the private sector or allotted for pilgrims to the Hajj [5]. The emergence of H5N1 and the A(H1N1)2009pdm virus, together with more country-specific data on the burden of disease, may have increased vaccine uptake in some countries, a pattern previously observed among European nations [6]. However, established vaccination guidelines and the extent of vaccine use have not been well documented. Given the shared global priority to improve access and affordability to influenza vaccines globally [7], as well as the 2012 Strategic Advisory Group of Experts (SAGE) on immunization recommendations for seasonal influenza vaccine, [8] our objectives were to compare country-specific published vaccine guidelines to the SAGE recommendations and report seasonal influenza vaccine sales data in Southeast Asia from 2008–2011.

## Materials and Methods

### Ethics Statement

The protocol was reviewed by the U.S. CDC and determined to be exempt from review by the Institutional Review Board. It was not reviewed by any other ethics board.

Selected economic and health indicators for the ten countries were obtained from publically available websites cited in the footnotes of Table 1. To obtain data on vaccine guidelines and vaccine sales data we electronically sent a standard three-page questionnaire to Ministries of Health in the ten Southeast Asian countries in the Association of Southeast Asian Nations [ASEAN: Brunei, Cambodia, Indonesia, Lao People’s Democratic Republic (PDR), Malaysia, the Republic of the Union of Myanmar (Myanmar), Philippines, Singapore, Thailand, and Vietnam]. We asked about formal guidelines for use of seasonal influenza vaccine, whether influenza vaccine was routinely used in the public sector (defined as purchased by the government and provided free of charge to citizens), the number of doses of vaccine imported from 2008 through 2011, and the presence of domestic capacity to produce human influenza vaccine. We also asked about receipt of purchased or donated monovalent A(H1N1)pdm09 vaccine in 2009 and 2010 and supplemented these data with publically available data about donated vaccine [9].

**Table 1:** Selected health, budgetary, and immunization statistics of the ten Southeast Asian countries surveyed.

Country	GNI per capita, international $^1^	Country incomeclassification^2^	Total expenditure onhealth as % GDP^1^	% routine^3^ childhoodvaccines funded bygovernment^4^	Under 5 childmortality rate per1000 live births^4^	% of deaths amongchildren <5 yearscaused by pneumonia^1^
Singapore	55,790	High	4.0	100	3	7% (5^th^)
Brunei	50,180	High	2.8	100	7	5% (5^th^)
Malaysia	14,220	Upper-middle	4.4	80	6	6% (5^th^)
Thailand	8,190	Upper-middle	3.9	100	13	9% (4^th^)
Indonesia	4,200	Lower-middle	2.6	100	35	14% (3^rd^)
Philippines	3,980	Lower-middle	3.6	100	29	16% (3^rd^)
Vietnam	3,070	Lower-middle	6.8	30	23	12% (4^th^)
Lao PDR	2,440	Lower-middle	4.5	6	54	19% (2^nd^)
Cambodia	2,080	Low	5.6	23	51	16% (3^rd^)
Myanmar	1,950	Low	2.0	0	66	17% (3^rd^)

GNI: Gross National Income; DGP: Gross Domesitc Product; USD: United States Dollars.

1 Total gross national income per-capita, total expenditure on health, percent of deaths among children <5 years caused by pneumonia and ranking of pneumonia as the cause of childhood mortality updated May 2012 and found at: levels for each country found at: http://www.who.int/countries/en/.

2 World Bank country classification can be found at: http://data.worldbank.org/about/country-classifications/country-and-lending-groups.

3 Routine childhood vaccinations are tuberculosis (BCG), diptheria, tetanus, pertussis (DTP), oral polio vaccine (OPV), hepatitis B, and measles-containing vaccine (MCV).

4 Percent of routine vaccinations funded by government and under 5-year mortality rates updated Oct 2012 and found at: http://apps.who.int/immunization_monitoring/en/globalsummary/countryprofileselect.cfm.

To better ascertain the total number of influenza vaccine doses sold to each country, we surveyed three multinational manufacturers. Globally, seven multinational manufactures produced 70% of seasonal influenza vaccine in 2009–2010 [10]. However, several of these large producers mainly serve a domestic market (not in ASEAN) and one has limited sales to Asia due to the high cost of the vaccine. It is estimated that the three manufacturers we surveyed supply >87% of the market share of influenza vaccine in the region (Mark Simmerman, Sanofi Pasteur Asia Pacific Medical Affairs, personal communication). The companies provided us with the number of doses of vaccines sold in the private sector in each country for the years 2008–2011 (data from one company were only through August 2011). Data were aggregated across the companies for confidentiality purposes, and data for Brunei/Malaysia and Cambodia/Lao PDR/Myanmar/Thailand/Vietnam were only provided in aggregate from some manufacturers. Sales figures are presented as rates per 100,000 persons using population data from the United Nations [11]. Data were entered in STATA version 11 (STATA, College Station, TX, USA).

## Results

The 10 ASEAN countries differed widely across the economic and health spectrum (Table 1). Two were high income countries, 6 middle income, and 2 low income. Total health expenditures as a percent of gross domestic product did not correlate with income classification. Under 5-year mortality rates were inversely correlated to income, with the highest rates in the low income countries. Geographically, these countries span from 9^o^ south to 22^o^ north of the equator. Of the ten countries contacted, nine (90%, all except Brunei) responded to our survey.

### Vaccine Sales in the Private Sector

Doses of vaccine sold to the private sector per 100,000 persons varied by country and year (Table 2). Among the four countries with non-aggregated data, the lowest rates (<200 per 100,000 persons) were consistently observed in Indonesia, a lower-middle income country. Singapore, a high income country, had the highest rates in 2008 (7034 per 100,000) and 2009 (12078 per 100,000). In 2010, Thailand, an upper-middle income country, surpassed Singapore (6910 vs. 6047 per 100,000) but Singapore was again highest in 2011 (7285 vs. 4943 per 100,000 persons). The aggregate rate of private sales in the 10 countries increased from 481 per 100,000 in 2008 to 954 per 100,000 in 2009 and the increase was sustained in 2010 and 2011 after the pandemic (Table 2).

**Table 2:** Influenza vaccine sales per 100,000 population; shaded areas are aggregate data from several countries. Dashed line (–) indicates data were not available.

	2008 trivalent seasonal vaccine		2009 trivalent seasonal vaccine		2010 trivalent seasonal vaccine		2009–2010 monovalent A(H1N1)pdm09 vaccine	2011 trivalent seasonal vaccine	
	Public	Private	Public	Private	Public	Private		Public	Private
Brunei	–	480	–	1465	–	1525	–	–	1030
Malaysia	–		–		–		1408	–	
Cambodia	0	367	0	832	0	1093	19189	0	916
Lao PDR	0		0		0		16136	0	
Myanmar	–		–		–		2027	–	
Thailand	752		3183		3439		2893	4051	
Vietnam	0		0		0		0	0	
Thailand†	752	856	3183	1869	3439	6910	2893	4051	4943
Indonesia	–	128	–	149	–	152	0	–	181
Philippines	0	1115	0	2551	0	1872	3646	1608	2305
Singapore	0	7034	0	12078	0	6047	25560	0	7285
TOTAL		451		954		898	1991*		898

† Private sales data in this row are from Thailand Food and Drug Administration.

* Excludes Brunei.

### Vaccine Sales in the Public Sector

Cambodia, Lao PDR and Vietnam, all low or lower-middle income countries, reported that no seasonal influenza vaccine was purchased by the government for use in the public sector during 2008 through mid-2011 (Table 2). The same was true for Singapore where influenza vaccines are routinely offered in public hospitals but are not free of charge. Malaysia (for use in healthcare workers), Indonesia and Myanmar purchased vaccine for use in the public sector but did not report the number of doses. The Philippines purchased seasonal vaccine beginning in 2011 (1608 per 100,000). In Thailand, the only other country to report public sales data, private sales increased by >300% from 520,000 doses in 2008 to 2.2 million doses in 2009, and continued to increase during 2010 (2.38 million doses) and 2011 (2.8 million doses, Table 2). In 2011, combined private and public sector sales in Thailand exceeded 7 million doses equal to 10,333 doses per 100,000 persons.

### Pandemic Vaccine Use

Seven countries in our study used A(H1N1)pdm09 monovalent vaccine during 2009 through 2010. All 6 lower-middle and low income countries were eligible for vaccine donated by multinational manufacturers and governments to WHO for global distribution, but only Cambodia, Lao PDR, Myanmar and the Philippines received A(H1N1)2009pdm monovalent vaccine [9]. Indonesia and Vietnam did not receive any A(H1N1)pdm09 vaccine despite being eligible for WHO donated vaccines. The three high and upper-middle income countries of Singapore, Malaysia and Thailand purchased A(H1N1)pdm09vaccine (Table 2). The greatest number of vaccine doses received or purchased per capita was in Singapore (25,560 per 100,000), followed by Cambodia (19,189 per 100,000) and Lao PDR (16,136 per 100,000).

### Vaccine Guidelines and Program

Current global recommendations for seasonal influenza vaccine include five groups: pregnant women, health care workers, children 5 to 59 months, the elderly, and persons with high-risk conditions. [8] Among these groups, pregnant women are listed as the most important group.

Five countries, Indonesia, Malaysia, Singapore, Thailand and Vietnam, all of which were high or middle income countries, had issued guidelines for the use of seasonal influenza vaccines (Table 3). Of the five countries with guidelines, two (40%) recommended vaccination for pregnant women, four (80%) for health care workers, three (60%) for young children, four (80%) for elderly persons and three (60%) for persons with underlying medical conditions. In addition, a number of other groups were recommended for vaccination (Table 3).

**Table 3:** Characteristics of seasonal influenza vaccine use among the nine Southeast Asian countries that responded to study survey**.**

Country	Public sector use (year of introduction)	Months of immunization administration	Vaccine guidelines	WHO recommended target groups [8]					Other target groups					
				Pregnantwomen	Health careworkers	Children 6–59months	Elderly	Persons withhigh riskconditions†	Institutionalizedindividuals	Medical care or hospitalization in previous year*	Children on long-term aspirin therapy	Pilgrims to the Hajj	Obesity	Household contact, caregivers of children or high risk
Cambodia	No	–	No											
Indonesia	Yes (2009)	Prior to the Hajj	Yes									✓		
Lao PDR	No	–	No											
Malaysia	Yes (1988)	Year round	Yes		✓		✓**		✓			✓		✓
Myanmar	Yes (2008)	Jun to Aug	No											
Philippines	Yes (2011)	May to Dec	No											
Singapore	No	Dec to Feb	Yes	✓(2/3 trimester)	✓	✓(6mo-5 years)	✓	✓		✓	✓			✓
Thailand	Yes (2004)	Jun to Aug	Yes	✓(3 trimester)	✓	✓ (6mo-2 years)	✓	✓	✓				✓ (≥100 kg)	
Vietnam	No	–	Yes		✓	✓(6mo-8 years)	✓	✓						

* Due to chronic metabolic diseases (including diabetes mellitus), renal dysfunction, haemoglobinopathies or immunosuppression (including by medication or HIV).

† High risk conditions: Malaysia (chronic cardiovascular, pulmonary, metabolic or renal disease, or who are immunocompromised); Philippines (chronic pulmonary or cardiovascular disorders); Singapore (diseases of the pulmonary or cardiovascular systems, including asthma); Thailand (chronic obstructive pulmonary disease, asthma, heart disease, stroke, kidney failure, cancer, diabetes, thalassemia, immunosuppression including persons infected with HIV); Vietnam (COPD, congenital heart disease, heart failure, diabetes, immunodeficiency).

** Only includes elderly with >1 more of the following chronic conditions: chronic cardiovascular, pulmonary, metabolic or renal disease, or who are immunocompromised.

Of the five countries with vaccine guidelines, three middle income countries (Indonesia, Malaysia and Thailand) had a seasonal influenza vaccination program in the public sector with dedicated funding (Table 3). Malaysia said that justification for vaccination was part of pandemic influenza preparedness. Both Malaysia and Thailand said that data on influenza morbidity and mortality were important factors in the decision to implement a seasonal influenza vaccination policy. Thailand also said that politics and pandemic preparedness were two additional factors. Indonesia said their decision was based on the requirements for the Hajj.

### In-country Vaccine Capacity

Three middle income countries, Indonesia, Thailand and Vietnam, reported developing in-country capacity for vaccine production. In 2007 and again in 2009, as part of a global initiative to transfer influenza vaccine technology to developing countries, WHO, supported by funds from the U.S. Biomedical Advanced Research and Development Authority (BARDA), awarded funds to Indonesia ($3.5 million), Thailand ($4 million) and Vietnam ($4.2 million) to complement local investments to develop domestic vaccine production capacities [12]. Thailand’s Government Pharmaceutical Organization is developing live-attenuated monovalent and trivalent vaccine using a Russian seed virus. Vietnam’s Institute of Vaccines and Medical Biologicals is producing inactivated monovalent influenza vaccine. BioFarma Indonesia acquired the technology to do fill and finish of seasonal influenza vaccine and is obtaining technology for up-stream processing and bulk production of antigen.

## Discussion

The Southeast Asian countries included in our study differ markedly in their demography, country income classification, and economic resources allocated for healthcare. Despite these differences, awareness of the importance of influenza prevention is increasing in the region as demonstrated through established influenza vaccine guidelines and increased influenza vaccine distribution in the majority of countries. Thailand, an upper-middle income country, and Singapore, a high income country, had the highest rate of vaccines purchased in the private sector since 2010. Indonesia, a lower-middle income country, reported the lowest rates of public sector influenza vaccine purchase. For the 10 countries combined, private sector sales during 2010 and 2011 (after the peak of the A(H1N1)pdm09 pandemic) exceeded pre-pandemic sales during 2008. It is too early to know if this positive effect of the A(H1N1)pdm09 pandemic on the demand for seasonal influenza vaccine will continue in future years suggesting a lasting influence. The only country reporting both private and public sector sales, Thailand, exceeded 10,000 doses per 100,000 persons in 2011. For comparison, in the 2010–2011 influenza season in the United States, 163 million doses of influenza vaccine were distributed for a crude rate of 52,312/100,000 [13], [14]. This 5-fold difference in the vaccine rate per capita between the two countries would likely be smaller if the denominator in Thailand were limited to the targeted risk groups. Nevertheless, it translates into 52% population coverage in the United States vs. 10% coverage in Thailand.

Vaccination guidelines varied substantially across ASEAN countries and in only two countries did guidelines reflect current global recommendations. [8] Most countries with national guidelines recommended vaccination of health care workers and elderly persons, consistent with data from Southeast Asia and other regions documenting the need to protect healthcare workers and their patients as well as elderly persons at increased risk for severe influenza [15], [16], [17]. In contrast, only two countries recommended vaccination for pregnant women, WHO’s highest priority risk group, and only three countries recommended vaccination for children aged 6–23 months, perhaps reflecting a need for more evidence on these risk groups in the region or a lack of resources to expand to these important risk groups. A recent study of influenza vaccine in pregnant women in Bangladesh demonstrated a 63% reduction in laboratory-confirmed influenza infections in infants of vaccinated mothers and better outcomes for gestational age and birth weight in infants [18], [19]. Local data on disease burden, seasonality, cost effectiveness, acceptability, coverage, and delivery strategies are needed to inform development and implementation of national vaccination strategies.

Receipt of A(H1N1)pdm09 pandemic vaccine correlated with income classification, in that poorer countries were eligible for donated vaccine and richer countries purchased vaccine. However, receipt of pandemic vaccine generally did not correlate with the scope of a country’s seasonal influenza vaccination policy. For example, Cambodia does not have seasonal vaccination guidelines but received a donation of 2.7 million doses of A(H1N1)pdm09 monovalent vaccine from WHO. In contrast, Vietnam, which has broad influenza vaccination recommendations, opted against using donated A(H1N1)pdm09 monovalent vaccine during the pandemic. The country cited their regulation to perform quality control testing of the vaccines prior to implementation as a rationale for declining the donation.

Despite the widespread adoption of influenza vaccine recommendations, the current amount of vaccine purchased in most countries does not meet the needs of the identified target groups. To address the growing need for more affordable and accessible influenza vaccines during a pandemic, the World Health Organization assisted eleven countries, including three middle income countries in Southeast Asia (Indonesia, Thailand, and Vietnam), in developing their domestic vaccine production capacity [12]. Indonesia’s BioFarma collaborated with Japan’s Biken Institute to develop local capacity. In July 2011, Thailand licensed a locally-produced, live-attenuated monovalent pandemic influenza vaccine and plans clinical trials on a trivalent influenza vaccine. Finally, Vietnam completed the construction of manufacturing plants for H5N1 and H1N1 vaccines in early 2011 and in April 2012 began a Phase I clinical trial for monovalent H1N1 vaccine. Although these countries are still several years away from commercial production of influenza vaccines that could meet regional needs, progress is clearly underway.

In the absence of locally available, affordable vaccine, lower income countries wanting to introduce vaccine have few options. In 2012, Lao PDR accepted 375,000 doses of seasonal influenza vaccine donated by Walgreens Corporation, a U.S.-based company [20]. This vaccine initiative represents an innovative new public-private partnership, the first of its kind in Lao PDR and perhaps a model for other countries in the region.

Our study has several limitations. First, because private sales data were only provided in aggregate for several countries, disparities in sales between these countries may have been hidden. Second, vaccine sales data are not the same as vaccine uptake as not all vaccines end up in a person’s arm. We did not account for vaccine wastage which would result in lower per capita rates. Third, our rate calculations did not account for the fact that children may have received two doses and this would have decreased the rates. Fourth, we did not limit the denominators to the target groups which would have increased the rates. Finally, private vaccine sales were underestimated because only three multinational vaccine manufacturers were surveyed; the Thailand FDA data are evidence of this.

We provide a summary of national influenza guidelines and influenza vaccination sales for ASEAN countries. Although vaccination sales remain generally low, consistent with global findings [21], we document a regional change towards increased vaccine sales. Advances in local vaccine production capacity in the coming years may further increase regional availability of vaccine. Public funding will be essential for national influenza vaccine programs to continue to develop and grow in Southeast Asia. Only Singapore and Thailand have vaccine recommendations that are consistent with global standards, suggesting that more work is needed to inform policy makers of data which support these important global recommendations.
